# Laparoscopic transabdominal extraperitoneal repair of lumbar hernia

**DOI:** 10.4103/0972-9941.16530

**Published:** 2005-06

**Authors:** A. Sharma, R. Panse, R. Khullar, V. Soni, M. Baijal, P. K. Chowbey

**Affiliations:** Department of Minimal Access Surgery, Sir Ganga Ram Hospital, New Delhi, India

**Keywords:** Laparoscopy, lumbar hernia, tension free mesh repair, transabdominal preperitoneal repair

## Abstract

Lumbar hernias need to be repaired due to the risk of incarceration and strangulation. A laparoscopic intraperitoneal approach in the modified flank position causes the intraperitoneal viscera to be displaced medially away from the hernia. The creation of a wide peritoneal flap around the hernial defect helps in mobilization of the colon, increased length of margin is available for coverage of mesh and more importantly for secure fixation of the mesh under vision to the underlying fascia. Laparoscopic lumbar hernia repair by this technique is a tensionless repair that diffuses total intra-abdominal pressure on each square inch of implanted mesh. The technique follows current principles of hernia repair and appears to confer all benefits of a minimal access approach.

## INTRODUCTION

Lumbar hernias are quite uncommon as compared to other ventral abdominal wall hernias. They may be congenital or acquired and anatomically divided into superior lumbar hernias (Grynfeltt's hernia, bounded by inferior edge of the 12th rib, lateral border of the erector spinae muscles and the lateral margin of the external oblique muscle) and inferior hernias (Petit's hernia, bounded by edge of the latissimus dorsi, the external oblique muscle and the superior edge of the iliac crest). More than 90% of the patients present with protrusion of the abdominal contents through the superior lumbar triangle. Acquired lumbar hernias are multifactorial, flank trauma and a previous flank incision are common etiologies. Though rare defects, lumbar hernias have 25% risk of incarceration and a >8% chance of strangulation.[1] Various repair techniques have been proposed such as simple anatomical repair, rotational musculofascial pedicle flap grafts, free grafts, fascial strip repair, and various synthetic mesh repairs. Failure of these repairs may be due to limited fascial strength, weakness of the surrounding tissues and difficulty in sewing the bony portion of the hernia boundaries. The principle behind the conventional tensionless prosthetic mesh repair of lumbar hernias seems to be reasonable but it requires a large incision to define the hernia edges and suturing the mesh around the hernia defect. Additional morbidity is due to retraction, compression of nerve endings leading to pain and a risk of postoperative hematoma, seroma, wound infection, or mesh infection.[2] Only a few cases of laparoscopic repair of lumbar hernia have been reported so far.[1][3]–[5]

Minimal access surgical techniques were applied for repair of lumbar hernia after the first successful repair by Burick and Parascandole in 1996.[3] Heniford et al.[1] described transperitoneal three port lateral position repair with PTFe patch secured with transabdominal polypropylene sutures. They fixed the mesh to the 12th rib superiorly and the iliac crest inferiorly by using orthopedic drill to create a hole and secure mesh to the bone by polypropylene sutures. Meinke et al.[4] described totally extraperitoneal videoendoscopic approach to lumbar hernia. They created a plane between transversalis muscle and the peritoneum by muscle splitting dissection through the lateral abdominal musculature and repaired the defect with PTFe mesh. Shekarriz et al.[5] performed laparoscopic transabdominal preperitoneal repair of lumbar incisional hernia in three patients and found it to be superior in terms of associated morbidity and convalescence over open approach.

We describe our technique of laparoscopic transabdominal preperitoneal repair of lumbar hernia. Modified flank position of the patient assists in surgery by displacing the intraperitoneal viscera medially towards the dependent side by gravity. Creation of peritoneal flap all around the hernial defect clearly defines the extraperitoneal contents, aids in complete reduction of the hernia, delineates the margins of hernial defect clearly and allows secure fixation of lower margin of the mesh to the lumbodorsal fascia. Reperitonealization by replacing the peritoneal flap over the mesh makes placement of the mesh entirely retroperitoneal.

## SURGICAL TECHNIQUE

**Figure 1:**
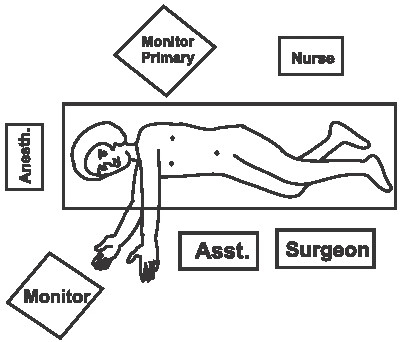
Operation theatre layout

**Figure 2:**
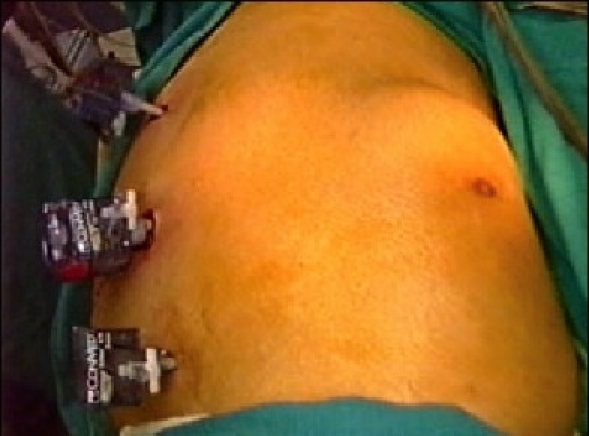
Position of ports

**Figure 3:**
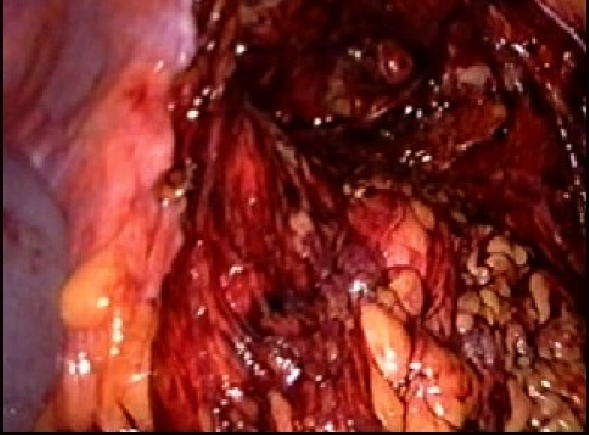
Lumbar hernia with extraperitoneal contents

**Figure 4:**
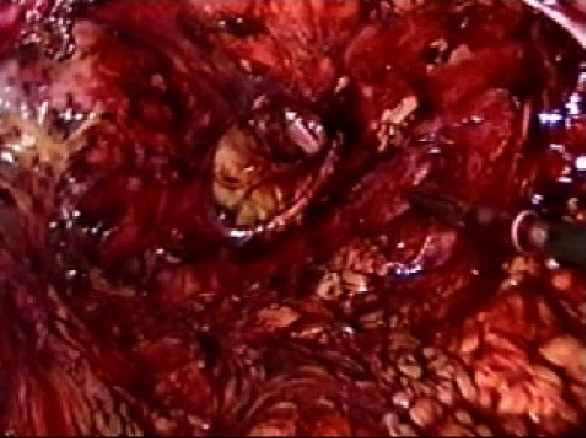
Lumbar hernial defect after reduction of contents

**Figure 5:**
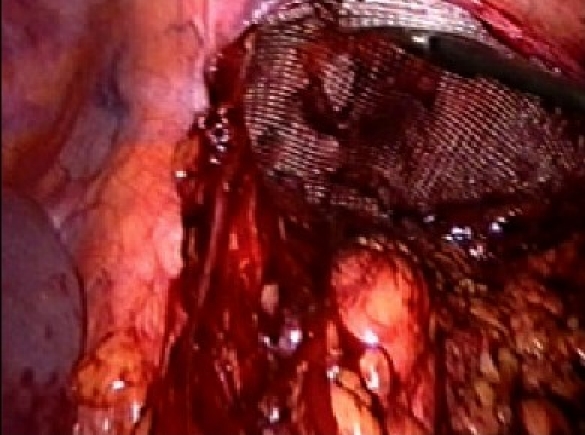
Polypropelene mesh covering the hernial defect

**Figure 6:**
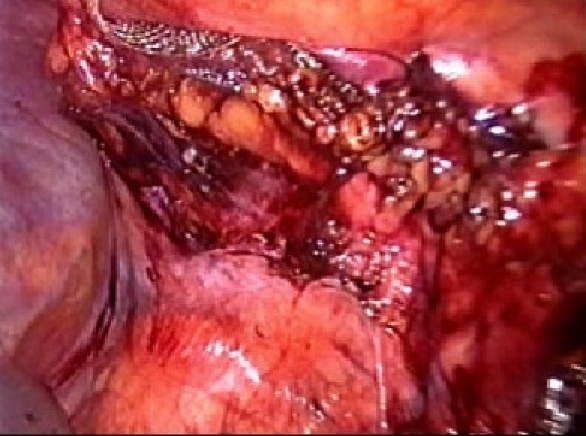
Reperitonealization of the mesh

The procedure is performed under general anesthesia. An orogastric tube is placed to decompress the stomach. The patient is placed in modified flank position with a 60° elevation of the side ipsilateral to the hernia and a lumbar roll in place. The bottom leg is flexed to 45° while upper leg is kept straight and a pillow placed between the legs. All pressure points are well padded and the patient is secured to the operating table with safety belts. The operating surgeon and the first assistant (camera person) are positioned facing the patient. The scrub nurse stands at the foot end of the operating table. The monitor is placed on the opposite side in line with the operative site [Figure 1]. Pneumoperitoneum is established by Veress needle through the Palmar's point (left subcostal midclavicular line at the lateral edge of the rectus abdominus muscle). A 5-mm safety shield trocar is inserted once the intra-abdominal pressure reaches 10 mmHg. Diagnostic laparoscopy is performed with a 5-mm, 30° telescope. An overview of the site of the hernia and intra-abdominal contents are made. Two working ports, a 10- and a 5-mm port are placed 10 cm from the hernia forming an arc of a circle with the hernia as the center [Figure 2]. Omental adhesions near the hernial defect are lysed. The contents of the hernial sac (omentum, ascending, or descending colon, small bowel) are reduced. The next step is to mobilize the colon. An incision is made along the lateral peritoneal fold at least 5 cm from the lower margin of the hernial defect. By this maneuver, the descending colon gets mobilized and is taken down with the lower peritoneal flap exposing the lower edge of lumbar hernia. Care is taken to prevent any injury to kidney, which is seen prominently at this step. The contents of the sac may comprise extraperitoneal fat, which is reduced gently and gradually [Figure 3]. After complete reduction of the contents, the margin of the hernial defect is clearly defined [Figure 4]. Lower peritoneal flap is developed so that at least five centimeters of clearance around the hernial defect is achieved. For small hernial defects, upper peritoneal flap may be created for five centimeters above the hernial defect. This helps in covering the mesh entirely making it completely extraperitoneal. But in case of large hernial defect requiring a large size mesh, creation of upper peritoneal flap is not required. “The main concern with” the peritoneal flap is to achieve secure fixation of the mesh to the underlying fascia. A half rolled 15 × 15 cm polypropylene mesh is placed over the defect by railroading the mesh with the help of nylon suture passed percutaneously through the center of the defect. The lower margin of the mesh is tucked in the groove lateral to the quadratus lumborum muscle and fixed to the lumbodorsal fascia with spiral tacks (ProTack™, Autosuture, Tyco Healthcare, US Surgicals, Norwalk, CT, USA). The mesh is fixed with spiral tacks at a distance of 1-2 cm with a ‘double crown technique.’ The mesh overlaps the defect for at least 4-5 cm in all direction [Figure 5]. Reperitonealization by replacing the peritoneal flap created earlier is performed by spiral tackers or by continuous intracorporeal suturing thus making the mesh entirely retroperitoneal [Figure 6]. The 10-mm port is closed under direct vision with port closure needle and skin clips applied. Pressure dressing is applied at the site of the hernia, which is removed on postoperative day 7.

The patient is allowed liquid diet and is ambulatory 2-3 h after the surgery. A normal diet is resumed on the day following the surgery and patient is discharged on oral medications. An abdominal binder is prescribed for support for 6 weeks.

## DISCUSSION

After the introduction of prosthetic repairs, recurrence rates following conventional (open) ventral abdominal wall hernia repairs has fallen to between 12.5 and 19%.[6] However, the large skin incisions, extensive dissection and mobilization, retraction, and bowel handling are responsible for significant wound morbidity postoperatively. Many of these patients have cardiopulmonary comorbidities that hamper early convalescence especially with prolonged surgery and pain associated with large incisions that restrict respiratory excursions postoperatively.

Currently, the Rives Stoppa approach appears to be the most promising open technique, with comparatively low recurrence rates.[7] However, this technique performed in tissue that is already of poor quality has led to a complication rate of up to 20% involving the wound, exposure and infection of the mesh, fistula formation, and other problems.[8] The laparoscopic approach is based on the Rives Stoppa technique and hence appears to confer advantages of both a low recurrence rate and low wound complication rate. We have been performing Laparoscopic ventral hernia repairs for more than a decade now with gratifying results.[9]

For lumbar hernias, modified flank position causes the intraperitoneal viscera to be displaced medially towards the dependent side by gravity. The gravity related traction exerted on the mesocolon results in anterior displacement of lateral peritoneal reflection. Creation of the peritoneal flap/envelope around the hernia helps to mobilize the colon away from the hernial defect.The hernial defect becomes more prominent and well defined after reflection of the peritoneal flap.

We prefer a polypropylene mesh for prosthetic repair as it has high intrinsic tensile strength, good memory and ‘see through’ nature of the mesh helps to cover the hernial defect with adequate margin. Covering the mesh by approximation of the peritoneal flap avoids its direct contact with colon or bowel and makes the mesh entirely retroperitoneal.

Extraperitoneal position of the mesh is advantageous and no bony anchorage is essential. Peritoneal coverage over the entire mesh provides additional security, and decreases potential postoperative adhesions. The weight of the intraperitoneal contents is an additional support to maintain the mesh in correct position in the early postoperative period.

Laparoscopic lumbar hernia repair by this technique is a tensionless repair. It follows the current principle of hernia surgery and is based on the sound physiological principle of diffusing the total intra-abdominal pressure on each square inch of the mesh implanted instead of the tenuous suture line -fascial interface. Mesh infection is rare as the ports are located well away from the hernial site.

Laparoscopic transabdominal preperitoneal mesh repair for lumbar hernia confers all the benefits of minimal access surgery to the patient and follows current principles of tensionless repair of ventral abdominal wall hernia.
